# Phenotypic Selection in *Halenia elliptica* D. Don (Gentianaceae), an Alpine Biennial with Mixed Mating System

**DOI:** 10.3390/plants11111488

**Published:** 2022-05-31

**Authors:** Xiaojuan Huang, Minyu Chen, Linlin Wang, Mingliu Yang, Nacai Yang, Zhonghu Li, Yuanwen Duan

**Affiliations:** 1 Key Laboratory of Resource Biology and Biotechnology in Western China, Ministry of Education, College of Life Sciences, Northwest University, Xi’an 710069, China; haungxiaojuan@mail.kib.ac.cn; 2 The Germplasm Bank of Wild Species, Institute of Tibetan Plateau Research at Kunming, Kunming Institute of Botany, Chinese Academy of Sciences, Kunming 650201, China; minyu1115@hotmail.com (M.C.); wanglinlin0328@mail.kib.ac.cn (L.W.); yangnc2022@126.com (N.Y.); 3 School of Ecology and Environmental Science, Yunnan University, Kunming 650091, China; yangml@mail.ynu.edu.cn; 4 Yunnan Lijiang Forest Ecosystem National Observation and Research Station, Kunming Institute of Botany, Chinese Academy of Sciences, Lijiang 674100, China

**Keywords:** phenotypic selection, selfing syndrome, mixed mating system, seed production, resource limitation, floral traits, spur length, *Halenia elliptica*

## Abstract

The transition from outcrossing to selfing is a common evolutionary trend in flowering plants, and floral traits change significantly with the evolution of selfing. Whether or not plant traits are subjected to selection remains an open question in species with mixed mating systems. We examined phenotypic selection in two populations of *Halenia elliptica* with different selfing rates. We found that the pollen–ovule ratio, seed size, plant height, spur length, and pollinator visitation rate in the population with the higher selfing rate were lower than those in the population with the lower selfing rate. Selfing provides reproductive assurance for populations when pollinator service is low, and the floral traits that are associated with selfing syndrome are evident in populations with a higher selfing rate but are subjected to weak selection in each of the two populations with different selfing rates. Directional selection for an early flowering time indicated that late blooming flowers could experience a risk of seed development in alpine environments, and for large plants, selection indicated that seed production could be limited by the available resources. The floral traits that are associated with pollinator attraction and specialization could be subjected to weak selection at the plant level as selfing evolves, and the selective pressures that are independent of pollinators might not change significantly; highlighting the selective biotic and abiotic pressures that shape the morphological traits of plant species and their independence from the mating system.

## 1. Introduction

More than 80% of flowering plants produce seeds by means of biotic pollen vectors [1], suggesting that pollinators are necessary for successful seed production in most plant species. However, the wide occurrence of limited pollen for seed production suggests that there might not be sufficient pollinators for many plant species [2,3,4]. The global decline in pollinator diversity could result in limited pollination for seed production [5,6,7,8,9,10], although several case studies have not found decreased seed production in wild plants in recent decades [11,12]. However, for outcrossing plants, the plant traits that are associated with pollinator attraction (e.g., plant height, flower time, flower size, flower tube depth, and flower number) and pollination efficiency (e.g., spur length) might be selected [13,14,15,16,17,18]. For example, pollinator-mediated selection may result in tall plants, plants with large flowers, more flowers, deep flower tubes, and long spurs, which are often reported in many plant species [14,17,19,20,21,22,23,24,25,26,27,28]. However, pollinator service often fluctuates across multiple populations and/or across years [29,30].

Reproductive assurance is considered to be a primary selective pressure for the evolution of autonomous selfing [31,32,33], allowing plant species to successfully produce seeds in habitats with low pollinator services [31,32,33,34,35,36,37]. Generally, in angiosperms, the shift from outcrossing to selfing is one of the most common evolutionary transitions and has occurred many times in the context of phylogenetic analyses [38]. The genetic advantage of gene transmission in selfing plants over outcrossing plants (3:2) also facilitates the evolution of autonomous selfing [39]. Accordingly, a shift from selfing to outcrossing is difficult in flowering plants [40] despite the counteracting disadvantages resulting from inbreeding depression and pollen (ovule) discounting. Thus, selfing is generally considered to be an evolutionary ‘dead end’ or ‘blind alley’ [41,42].

In floral traits, selfing plants often show more significant differences than outcrossed plants. Firstly, this is because the traits that are associated with pollinator attraction would be redundant in selfing plants, so those traits would be significantly reduced [32,43,44,45,46]. For example, plant size and flower size represent similar traits in pollinator attraction, and according to a large taxonomic survey on angiosperms, plants that are large and that have large flowers can enhance pollinator attraction, leading to a negative correlation between flower size and the selfing rate [44,47]. Secondly, as a consequence of a reduced number of resources being allocated to flowers for pollinator attraction in selfing plants, the resources would be reallocated to other flower organs [48]. In selfing species, ovule production is generally higher than it is in paired outcrossing sister species [49], which increases fitness through the female function in selfing plants. Therefore, the pollen–ovule ratio is smaller in selfing plants than it is in outcrossing species [49,50,51], which can also be attributed to higher pollination efficiency in selfing plants than in outcrossing ones [50]. In addition, spur length is considered to be a key innovation in speciation [52], and it is correlated to pollination efficiency. Directional evolution towards the long spurs in *Aquilegia* that are associated with shifts in pollinators with long tongues was found to drive speciation in this genus [52,53,54]. The suite of morphological traits accompanying shifts to self-fertilization comprises ‘selfing syndrome’ [32,44], and selfing syndrome is more evident in plant species/populations with high selfing rates [55,56,57], indicating the selection of selfing syndrome at the species/population level. However, it largely remains unclear as to whether or not floral traits are subjected to different phenotypic selection in mixed-mating species in relation to pollinator attraction (flowering date, plant height, and flower tube depth) and pollination efficiency (spur length), which could be resolved through comparisons of phenotypic selection in populations with different selfing rates. In this study, we investigated the phenotypic selection of morphological traits in *Halenia elliptica* D. Don (Gentianaceae) in two populations with different selfing rates. *Halenia elliptica* is a biennial herb with a wide distribution in western and northern China [58]. The phenotypic selection of floral traits was studied via comparisons between netted and control flowers. This species produces seeds via autonomous selfing and outcrossing. However, the outcrossing rate of *H. elliptica* varies significantly across a latitudinal gradient (0.37–0.85), with high outcrossing rates in low latitude populations and low outcrossing rates in high latitude populations [59]. For outcrossing plants, increasing the plant traits in relation to pollinator attraction (e.g., plant height, flowering date, flower size, flower number, and flower tube depth) and pollination efficiency (e.g., spur length) might be selected, and we expect that the plants in the high selfing rate population favor a lower plant height, a smaller flower size, shorter flower tube depth, and a shorter spur length. In addition, we also expected that selfing weakens the phenotypic selection of floral traits in relation to the pollination efficiency. Specifically, we addressed the following questions: (1) what are the differences in the morphological traits and pollinator visitation between the two populations? (2) What are the differences in the strength of phenotypic selection on morphological traits in relation to pollinator attraction and pollination efficiency between the two populations?

## 2. Results

### 2.1. Flower Traits, Pollinator Observation and Seed Size

**Figure 1:**
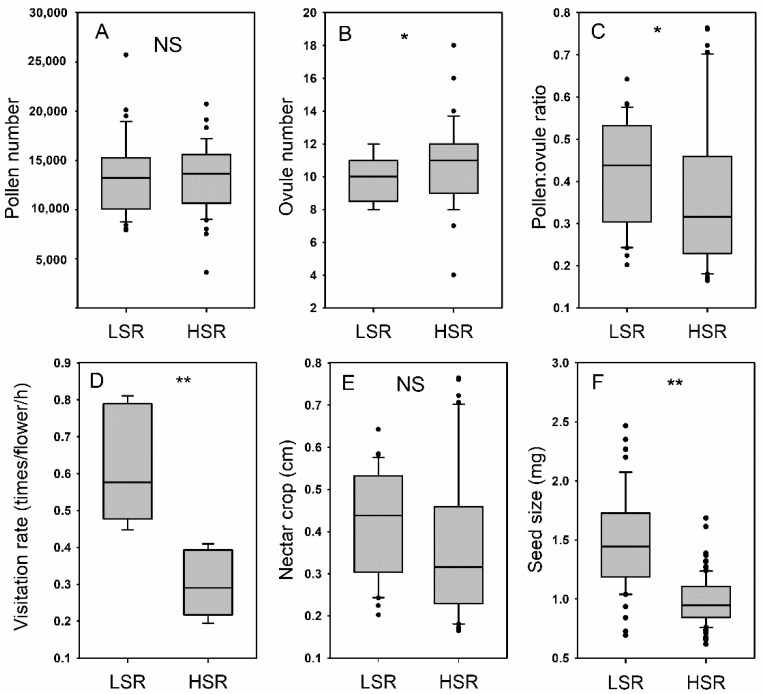
Pollen number (**A**), ovule number (**B**), pollen–ovule ratio (**C**), pollinator visitation rate (**D**), nectar crop (**E**), and seed size (**F**) of *Halenia elliptica* in a population with a low selfing rate (LSR) and a high selfing rate (HSR). Seed production comparisons are shown in Table 1. **, *p* < 0.01; *, *p* < 0.05; NS, *p* > 0.05.

No significant differences were observed in the pollen number between the two populations with different selfing rates (Figure 1A), but the plants from the LSR (low selfing rate) population in Lijiang had a lower ovule number than the plants in the HSR (high selfing rate) population in Huangyuan (Figure 1B), resulting in the LSR population having a higher pollen–ratio than the HSR population (Figure 1C). Both bumblebees and honeybees were identified as the most frequent pollinators in the two populations, accounting for more than 90% of the total visits. Bumblebees and honeybees demonstrated similar visiting behaviors on the flowers, collecting nectar from the spurs of *H. elliptica*. The pollinator visitation rate in the LSR population was significantly higher than it was in the HSR population (Figure 1D), although no significant differences were observed in the nectar volume between the two populations (Figure 1E). In the LSR population, the seed size was significantly larger (Figure 1F).

### 2.2. Phenotypic Selection on Morphological Traits

**Figure 2:**
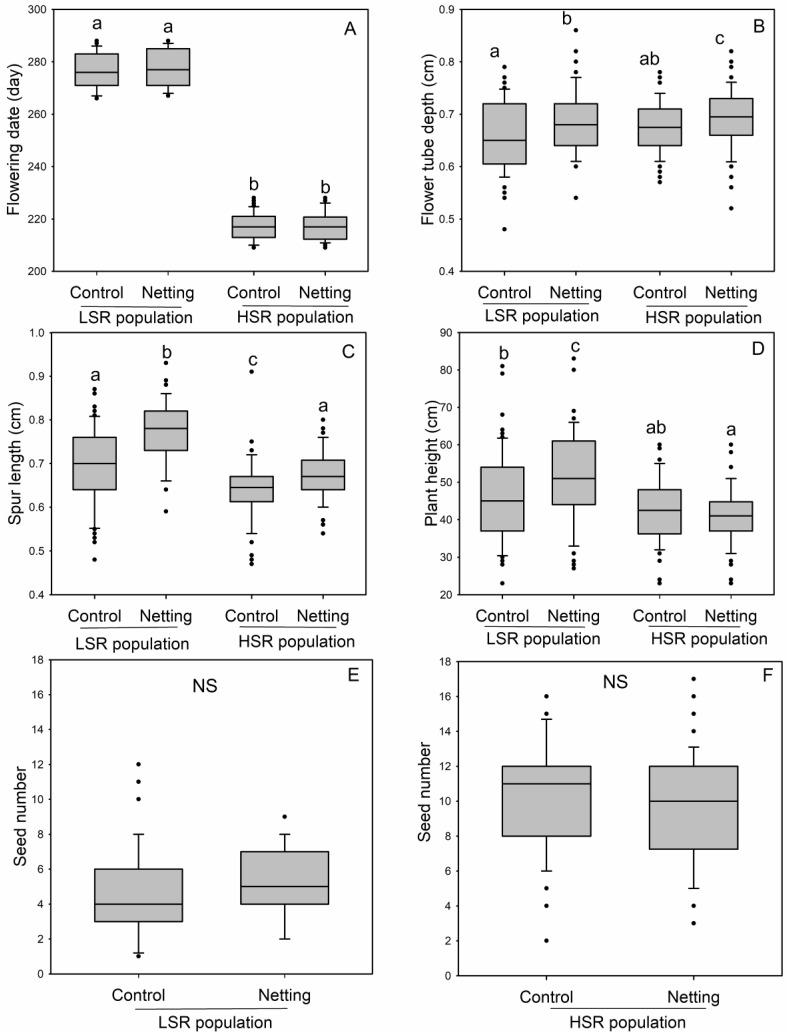
Flowering date (**A**), flower tube depth (**B**), spur length (**C**), and plant height (**D**) between *Halenia elliptica* netted and control flowers in the low selfing rate (LSR) and high selfing rate (HSR) populations. Seed numbers for the netted and control flowers are also shown for the populations with low (**E**) and high selfing rates (**F**). The results of the comparisons are shown in Table 1. Different letters beside the bars indicate significant differences at the *p* = 0.05 level.

Flowering date, flower tube depth, spur length, and plant height varied between populations and treatments (Figure 2 and Table 1). The plants in the LSR population flowered later than those in the HSR population, and no significant differences were observed in terms of the flowering date between the netted and control flowers within the two populations (Figure 2A, Table 1). The flower tube depth of netted flowers was higher than that of the control flowers within the populations, and no significant differences were observed in the flower tube depth between the populations (Figure 2B, Table 1). The spur length of the netted flowers was higher than that of the control flowers within both populations, and the spur length of the flowers in the LSR population was also higher than it was in the HSR population (Figure 2C, Table 1). The LSR population had higher plant height than the HSR population, and no significant differences were observed in the plant height between treatments (Figure 2D, Table 1). Collectively, the floral traits were significantly affected by the population and treatment, and the flowering date and plant height were significantly affected by the population (Table 1). The seed number was significantly lower in the LSR population than it was in the HSR population (Figure 2E, F). In each population, no significant differences in the seed number were found between the netted and control flowers, determining that cross-pollination is not necessary for seed production. The treatment type and population also showed an interaction effect on the seed number (Table 1).

In both populations, most of the floral traits were positively correlated with each other; however, the flowering date was negatively correlated with the flower tube depth and spur length in the HSR population (Table 2). In the LSR population, the flowering date was negatively related to the flower tube depth and was positively related to the plant height, but no significant relationship was found between the flowering date and the spur length (Table 2). The flower tube depth was positively related to the spur length, and no significant relationship was found between the flower tube depth and the plant height or between the spur length and the plant height (Table 2). In the HSR population, the flowering date was also negatively related to the flower tube depth, and no significant relationship was found between the flowering date and the spur length or between the flowering date and the plant height (Table 2). In contrast, no significant relationship was found among the flower tube depth, spur length, and plant height (Table 2).

**Figure 3:**
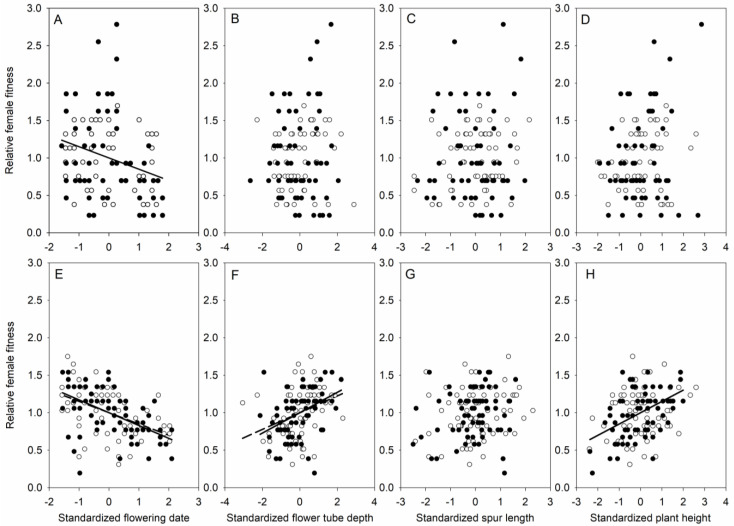
Linear selection differentials for the flowering date (**A**,**E**), flower tube depth (**B**,**F**), spur length (**C**,**G**), and plant height (**D**,**H**) in the control (filled circles and line, *p* < 0.05) and netted (open circles and dotted line, *p* < 0.05) flowers from two populations with low (**A**–**D**) and high (**E**–**H**) selfing rates.

In the HSR population, the linear selection differentials favored an earlier flowering date and a higher flower tube depth in both the control and netted treatments, as well as a higher plant height in the control treatments (Figure 3 and Table 3). In the LSR population, most of the studied flower traits were not selected in neither the control nor the netted treatments, with the exception of the earlier flowering date, which was significantly selected in the control treatments (Figure 3 and Table 3). In addition, spur length was not selected in neither the control nor the netted treatments in both the LSR and HSR populations (Figure 3 and Table 3). When all of the measured traits were included, earlier flowering date and higher plant height were selected for both control and netted treatments in the LSR and HSR populations, based on the linear selection gradients, with the exception of flowering date in the LSR population in the netted treatments (Table 4). Both flower tube depth and spur length were not selected for neither the control nor the netted treatments in the LSR and HSR populations (Table 4).

## 3. Discussion

### 3.1. Variation of Morphological Traits

Reproductive assurance is an important explanation for selfing evolution in plants [60]. Spatial variation in pollinator visitation could drive the evolution of floral traits and increase selfing rates in mixed mating species when pollinators are scarce. Our previous studies showed that in *Halenia elliptica*, the selfing rate increased as the latitude increased [59,61]. In the present study, we found a reduced pollinator visitation rate in the HSR population around Huangyuan compared to the LSR population around Lijiang (Figure 1D). The convergent loss of outcrossing-associated traits in plants with predominant selfing are phenotypic characteristics of selfing syndrome [44], and there is a general trend towards small, inconspicuous flowers with stigmas and anthers that are in close proximity to each other, limited pollen production, low pollen–ovule ratios, and a loss of nectar production in selfing populations [42]. By comparing the flower traits in high and low selfing populations, we found that the pollen–ovule ratio and spur length were lower in the HSR population. Therefore, our results only partially support these selfing syndrome trends. (Figure 1 and Figure 2).

Reductions in the pollen–ovule ratios are commonly due to reductions in pollen instead of increases in the number of ovules [62]; however, in *H. elliptica,* the reduction in the pollen–ovule ratio was attributed to increased ovule production in the HSR population instead of a decreased pollen number (Figure 1A–C). Since no pollen limitation occurred in the HSR population (Figure 1 and Table 1), the resource reallocation between plants enhanced female function [62,63]. For example, the seed size decreased, and the seed number increased in the HSR population, compared to in the LSR population (Figure 1 and Table 1), which could be the result of the seed number–size trade-off and resource reallocation [62,64]. For plants with capsules that depend on gravity for seed dispersal, small seeds can be dispersed more easily than large seeds, although germination rate and/or seedling survival rate might also reduce for small seeds. Therefore, the reduced seed size in the *H. elliptica* plants in the HSR population could be induced by the increased seed production and the resulting increase in sibling competition [65,66]. The decrease in seed size and the increase in seed number may be due to the adaptation to various factors, such as climate, seed predators, etc. However, when the different populations were undergoing the different treatments, no significant differences were observed in the seed numbers (Figure 2). The results confirmed that in *H. elliptica,* seed production mainly depends on selfing.

Although the HSR population received fewer pollinator visits and had a smaller spur length compared to the LSR population, no significant differences were observed in nectar production between the two populations. There is a high cost for nectar production in most species, and it should not be selected in high-selfing plant species or populations. For plant species with spurs, nectar robbing frequently occurs [67], and nectar thieves could maintain nectar production through the continuous collection of nectar [68]. In the fields, we observed small holes on the spurs of some individuals of *H. elliptica* plants, which could be used by nectar robbers and that could maintain nectar production.

### 3.2. Phenotypic Selection on Morphological Traits

The phenotypic selection of floral traits can be examined by measuring the relationships between female fitness and floral traits [69]. Excluding the netted flowers in the LSR population, an early flowering date was strongly selected in the two *H. elliptica* populations (Table 3 and Table 4). These results are in accordance with recent studies indicating that early flowering plants are favored in many flowering plant species [70]. There are many reasons for early flowering, such as population size, plant size, flowering duration, growing season length, and other environmental factors [17,70]. For example, regardless of the altitude in alpine ecosystems, seed production can be limited by there being a lower mating probability among late-flowering individuals [71], and the limited time for seed development because of the short growing season and the low temperature in the later growing season [72].

Plant height is an indicator of total amount of resources invested in above-ground biomass and mirrors the resources that are available to the plants within one species. In plants that are capable of selfing, seed production would not be limited by pollen availability and would only be limited by resource availability [68]. Therefore, it would be expected that large plants that have more invested resources would produce more seeds than small plants with less resource investment [14,15]. Our results strongly support this prediction since we found positive phenotypic selection for plant height in the two *H. elliptica* populations with different selfing rates. However, the effects of variations in the phenotypic selection gradients on plant height could be attributed to interactions with other correlated traits (such as flower tube depth) and pollinators in different populations. Plant height and flower tube depth are likely to affect the probability of pollinator visitation, and a positive correlation on plant height and flower tube depth has been documented to be due to the synergistic effect of these two traits in pollinator attraction [16]. At the same time, there may be a greater presence of pollinators in low-latitude populations. We predicted that the differences in plant height may also be different due to differences in latitude, which our study did not confirm.

The phenotypic selection of floral traits was studied by comparing netted and control flowers. Netted is mainly used to isolate pollinators. Differential phenotypic selection was significant in the control flowers in the HSR population, but no significant selection was documented for plant height in the netted flowers in the HSR population, in the control or in the netted flowers in the LSR population (Table 3); indicating that the phenotypic selection of plant height is mediated by agents other than pollinators in the LSR *H. elliptica* population. The plants in the LSR population were taller than those in the HSR population, which may explain why plant height is more critical for seed production in the former population. It could also be because they grow in a relatively shaded environment in the nets. No significant selection of flower tube depth was documented in the two populations (Table 3), but there were significant selection differentials in the HSR population, due to indirect selection via correlated traits, such as plant height. Taken together, the present results suggest that flower tube depth strongly influences pollination success in the population of *H. elliptica* with high selfing rates. In contrast, although we found a reduction of flower spur length in populations with a high selfing rate, flower tube depth did not change significantly across the two populations with different selfing rates, suggesting that the strength of selection may vary with plant habitat.

The evolution of floral traits in relation to pollination efficiency is generally associated with their functional pollinator groups and the evolution of spur length [73], and changes in key floral traits are often involved in pollinator shifts [52,74], which could further contribute to reproductive isolation and the resulting speciation [75]. In the deceptive orchid *Dactylorhiza lapponica,* there is directional selection for flowers with long spurs [15], indicating the importance of long spurs in maximizing female fitness. The similar selection of long spurs is also found in the rewarding orchid *Gymnadenia conopsea* [14]. In contrast, we did not find any significant selection differentials or gradients for spur length via female fitness in the two populations of *H. elliptica*, although changes in spur length were observed in the netted and control treatments, because the netted treatments reduced the light collected by the plants and increased the plant height, flower tube depth, and spur length [76,77]. A possible explanation for this might be that *H. elliptica* can produce seeds via both selfing and outcrossing, and thus the pollinator-mediated selection of spur length could be weak due to there being less dependence on pollinator abundance.

In summary, we examined plant traits and the phenotypic selection of floral traits in two populations of *H. elliptica* with different selfing rates and found a reduced pollen–ovule ratio, small seed size, a low pollinator visitation rate, and a short spur length in the HSR population. The strong selection of an early flowering time could be a common trait in plant species in alpine/arctic environments with a short growing season. The strong phenotypic selection of taller plants indicated limited seed production resources in *H. elliptica*. However, we did not find significant phenotypic selections for spur length in the two populations, suggesting that the floral traits that are associated with pollination efficiency are subjected to weak phenotypic selection in *H. elliptica*. The present results suggest that visual cues, such as plant height and flower tube depth, strongly influence pollination success, and that selfing could weaken the phenotypic selection of floral traits in relation to pollination efficiency in plant species with mixed mating systems.

## 4. Materials and Methods

### 4.1. Species and Populations

In order to comprehensively understand the study material, we have described it in detail. There are approximately 100 species of *Halenia* (Gentianaceae), which are characterized by flower spurs compared to other gentians [58]. This genus originated in East Asia, and migrated into and diversified in America [74]. In China, there are only two *Halenia* species, which are known as *H. corniculata* (L.) Cornaz and *H. elliptica* D. Don. *Halenia elliptica* is a biennial herb that is widely distributed in western and northern China [58]. The height of *H. elliptica* varies between 15 cm and 90 cm, and there are several cymose inflorescences on each plant. There are usually more than 10 flowers on each plant, and the total number of flowers strongly depends on the plant size, with larger plants having more flowers. The corolla is blue or purple and forms four tubes with a narrow opening on the bottom, and nectar is produced in the spurs (Supplementary Figure S1A). A single flower remains open for approximately 3–4 days. *Halenia elliptica* can produce seeds via autonomous selfing or with the aid of pollinators [78]. Bees, including bumblebees, solitary bees, and honeybees, are the main pollinators [59]. Each capsule produces about 12 seeds [78].

There are two varieties of *H. elliptica* that are categorized based on flower size and spur morphology, which are known as *H. elliptica* var. *elliptica* D. Don and *H. elliptica* var. *grandiflora* Hemsl. However, we found post-pollination reproductive isolation between the two varieties, and, thus, the two varieties of *H. elliptica* should be revised as being two separate species (*H. elliptica* and *H. grandiflora*) [79]. In this study, we selected two *H. elliptica* populations with different outcrossing rates to examine the differences in the phenotypic selection of morphological traits. The two study populations were located at Huangyuan (36°31′43″ N, 101°15′48″ E, 3072 m, Qinghai province) and Lijiang (26°59′56″ N, 100°11′59″ E, 2622 m, Yunnan province). The two populations were located in alpine meadows, and there were more than 3000 plants in each group. In the population in Lijiang, the outcrossing rate was approximately 0.87 ± 0.07 [59] and this population was considered to have a low selfing rate (LSR). In contrast, in the Huangyuan population, the outcrossing rate was approximately 0.44 ± 0.04 [59] and this population was considered to have a high selfing rate (HSR). All of the experiments were performed in the two populations between 2017 and 2018. All the observations and experiments were carried out in the wild populations in their natural habitats.

### 4.2. Flower Traits, Pollinator Observation and Seed Size

In each of the two populations, to make the two populations comparable and to reduce position-associated differences in the floral traits [80,81], we collected one top bud from each plant in 2017, collecting a total of 30 top buds (a top bud refers to the bud at the top of a plant’s main stem). The buds were kept separated in FAA solution (formalin: acetic acid: alcohol, 5:5:90 by volume). In the laboratory, ovule number was determined for each flower using a stereoscopic microscope. Four anthers were crushed in a centrifuge tube with FAA solution inside, and three drops of detergent were added to the tube to make the solution a full suspension. We added FAA solution to each tube for a constant volume of 0.5 mL, and in each tube, 5 uL replicates were observed using a microscope to determine the pollen number. The total pollen number and the pollen–ovule ratio, were calculated for each flower. To quantify the nectar crop of the two populations, we selected 30 newly opened flowers from the tops of different plants in each of the two populations and netted them from 9:00 to 10:00 in the morning. From 9:00 to 10:00 the next day, we collected the nectar in the four spurs of each flower using a capillary and measured the length of the nectar in the capillary.

At peak flowering in both populations, we selected three to five plants and counted the total number of open flowers on each plant. Then, we observed the flower visitors to these plants and determined whether the visitors were pollinators by observing whether the insects could touch stigma and/or anthers while collecting the nectar in the spurs. All observations were performed from 10:00 to 18:00 on sunny days, and we would stop observations if it became cloudy or rained when pollinator activity was low. Total observations were performed over five days, with the collection period amounting to 30 h in both populations. The average visitation frequency was then calculated.

To determine the differences in the seed size between the two populations, we collected 100 ripe fruits from the tops of different plants and determined the number of seeds in each fruit in the laboratory. All of the seeds were put in the drying room to determine a constant weight, and the seeds in each fruit were weighed using a balance to a minimum weight of 0.0001 g. The weight per seed was then calculated as the seed size.

### 4.3. Phenotypic Selection of Morphological Traits

To quantify the phenotypic selection of morphological traits, plants with visible flower buds were chosen at random and individually tagged. In 2018, we labelled 200 plants in each of the two populations and separated them into two groups. One group of 100 plants was netted to exclude pollinators, and the other group of 100 plants were treated as a control. The netted treatment involved wrapping the whole plants at the bud stage with mosquito net bags (Supplementary Figure S2). This meant that the phenotypic selection in these netted plants was the result of abiotic factors. Previous research revealed that shade can lead to taller plants, a larger plant mass [77], and an increased flower diameter [78]. In our study, the netted treatment was equivalent to the shade treatment, which reduced the light that could be absorbed by the plants, increasing the flower size, flower tube depth, and spur length (Supplementary Figure S1B,C). The study populations were visited approximately every four days throughout the flowering period, and we labelled the top bud on each plant during each visit until a total of 200 plants were labelled. For each plant, when the labelled top bud opened, we recorded the date that the flower opened (reported as the day of the year) and the plant height (distance from the ground to the top flower). We also measured the flower tube depth (petal length from the bottom to the top) of each labelled flower, using digital callipers. To measure the curled spur length of each flower, we fit a piece of string from the spur tip to the bottom of the corolla tube and then measured the length of the string. All of the measurements pertaining to flowering date and flower tube depth, spur length, and plant height were performed on the day that the labelled flowers opened. Fruits were collected from all of the labelled flowers for seed number measurements when the fruits were ripe, but not dehiscence. Here, we used the number of seeds in one fruit as the measure of female fitness. Meanwhile, sheep grazing decreased the sample size for each group, and the sample size reduced to 61 fruits for the control plants and 59 fruits for the netted plants in the LSR population in Lijiang and to 72 fruits for the control plants and 68 fruits for the netted plants in the HSR population in Huangyuan.

### 4.4. Statistical Analysis

The normality of the residuals for each of the parameters was performed for each parameter, and log-transformation was performed on the parameters that did not conform to a normal distribution. To examine the differences in reproductive traits, such as the pollen number, ovule number, pollen–ovule ratio, pollinator visitation rate, nectar crop, and seed size between the two populations with different selfing rates, we fitted generalized linear models (GLMs) and used the population as the fixed factor. All the experimental data are listed in supplementary Tables (Supplementary Tables S1 and S2).

For the data collected for phenotypic selection, the flowering date was transformed to the day of the year by calculating the number of days from January 1st, 2018 [18]. We first performed bivariate correlations among all of the measured traits. To test the differences in the flowering date, plant height, flower tube depth, spur length, and seed production, we fitted the GLMs and used the population and treatment as fixed factors. Then, for each treatment in each population, each group of data including the flowering date, plant height, flower tube depth, and spur length were standardized with a mean of 0 and standard deviation of 1, and the seed numbers for each group were transformed to the relative fitness by dividing the seed number for each fruit with mean seed number of the corresponding group [82,83]. Using relative fitness as a response variable for each group, we performed linear regression between relative fitness and each measured trait to examine the linear selection differentials, which estimated both the direct selection of a trait and indirect selection via correlated traits [84,85]. Furthermore, we performed linear regression between the relative fitness and all of the measured traits to examine the linear selection gradients, which estimated the direct selection of each trait independently of the selection of any of the other measured traits [86]. We also performed quadratic regression between the relative fitness and all of the measured traits to depict disruptive or stabilizing selection, but no cases of statistically significant nonlinear selection were detected. Therefore, we did not present the results of quadratic selection here. All analyses were performed with SPSS 16.

## 5. Conclusions

In the present study, we examined natural selection in two populations of *H. elliptica* with different selfing rates. The pollen–ovule ratio, seed size, spur length, and low pollinator visitation rate in the population with a high selfing rate were smaller than those in the population with a low selfing rate, but flower tube depth did not change significantly in the two populations with different selfing rates. Selfing provides reproductive assurance for populations when pollinator service is low, and the floral traits that are associated with selfing syndrome are evident in populations with a high selfing rate. However, the floral traits that are associated with pollinator attraction and specialization could be subjected to weak selection during the evolution of selfing in each of the two populations, and the selective pressures on flower traits that are independent of pollinators might not change significantly across multiple populations. These results suggest that biotic and abiotic factors play a role in the selective pressures that shape the morphological traits of plant species.

## Figures and Tables

**Table 1:** Effects of treatment (netting and control), population (low selfing rate and high selfing rate), and their interaction on the flower date, flower tube depth, spur length, plant height, and seed production using a generalized linear model in *Halenia elliptica*. Significant effects are shown in bold.

Source	Flowering Date (Day of Year)			Flower Tube Depth (cm)			Spur Length (cm)			Plant Height (cm)			Seed Production		
	Wald χ2	d.f.	Sig.	Wald χ2	d.f.	Sig.	Wald χ2	d.f.	Sig.	Wald χ2	d.f.	Sig.	Wald χ2	d.f.	Sig.
Treatment	0.35	1	0.56	**11.26**	**1**	**<0.01**	**35.58**	**1**	**<0.01**	0.96	1	0.33	0.19	1	0.66
Population	**6500.42**	**1**	**<0.01**	2.73	1	0.10	**65.15**	**1**	**<0.01**	**24.37**	**1**	**<0.01**	**219.72**	**1**	**<0.01**
Treat. × Pop.	0.07	1	0.79	0.02	1	0.88	**5.25**	**1**	**0.02**	**8.36**	**1**	**<0.01**	**5.59**	**1**	**0.02**

**Table 2:** Phenotypic correlations among the flowering date, flower tube depth, spur length, and plant height in the populations of *Halenia elliptica* with a low selfing rate (LSR) from Lijiang (above diagonal) and a high selfing rate (HSR) from Huangyuan (below diagonal). One and two asterisks indicate the significant relationships at the 0.05 and 0.01 levels.

	Flowering Date (Day of Year)	Flower Tube Depth (cm)	Spur Length (cm)	Plant Height (cm)
Flowering date (Day of Year)	−	−0.19 *	0.03	0.36 **
Flower tube depth (cm)	−0.24 **	−	0.23 *	<0.01
Spur length (cm)	−0.01	0.07	−	0.11
Plant height (cm)	0.09	0.06	0.02	−

**Table 3:** Phenotypic linear selection differentials (±SE) for the flowering date, flower tube depth, spur length, and plant height via female fitness in netted and control flowers from two *Halenia elliptica* populations with different selfing rates. Significant selection differentials are shown in bold.

Traits	Low Selfing Rate Population				High Selfing Rate Population			
	Control		Netting		Control		Netting	
	B	Sig.	B	Sig.	B	Sig.	B	Sig.
Flowering date (Day of Year)	**−0.15 ± 0.08**	**0.05**	−0.06 ± 0.05	0.22	**−0.17 ± 0.03**	**<0.01**	**−0.15 ± 0.04**	**<0.01**
Flower tube depth (cm)	0.04 ± 0.08	0.63	0.07 ± 0.05	0.19	**0.14 ± 0.03**	**<0.01**	**0.11 ± 0.04**	**<0.01**
Spur length (cm)	0.01 ± 0.08	0.96	0.08 ± 0.05	0.09	0.07 ± 0.04	0.06	0.06 ± 0.04	0.12
Plant height (cm)	0.13 ± 0.08	0.09	0.08 ± 0.05	0.13	**0.15 ± 0.034**	**<0.01**	0.06 ± 0.04	0.15

**Table 4:** Phenotypic linear selection gradients (±SE) for the flowering date, flower tube depth, spur length, and plant height via female fitness in netted and natural control flowers from two *Halenia elliptica* populations with different selfing rates. Significant phenotypic linear selection gradients are shown in bold.

Traits	Low Selfing Rate Population				High Selfing Rate Population			
	Control		Netting		Control		Netting	
	B	Sig.	B	Sig.	B	Sig.	B	Sig.
Flowering date (Day of Year)	**−0.24 ± 0.08**	**<0.01**	−0.09 ± 0.05	0.10	**−0.16 ± 0.03**	**<0.01**	**−0.15 ± 0.04**	**<0.01**
Flower tube depth (cm)	−0.04 ± 0.11	0.75	0.01 ± 0.06	0.87	0.05 ± 0.03	0.10	0.05 ± 0.04	0.26
Spur length (cm)	0.03 ± 0.11	0.78	0.08 ± 0.05	0.15	0.04 ± 0.03	0.18	0.04 ± 0.04	0.40
Plant height (cm)	**0.21 ± 0.08**	**0.01**	**0.12 ± 0.05**	**0.03**	**0.15 ± 0.03**	**<0.01**	**0.08 ± 0.03**	**0.03**

## Data Availability

Not applicable.

## Supplementary Materials

The following supporting information can be downloaded at: https://www.mdpi.com/article/10.3390/plants11111488/s1, Supplementary Table S1: The experimental deta of Lijiang. Supplementary Table S2: The experimental data of Huangyuan. Supplementary Figure S1: Wrapping the whole plant at the bud stage with mosquito net bags. Supplementary Figure S2: The morphological characteristics for *Halenia elliptica*. (A) Plant height, (B) spur length, (C) flower tube depth.
