# Supplementary material for: Phenotypic Selection in Halenia elliptica D. Don (Gentianaceae), an Alpine Biennial with Mixed Mating System
Source: Plants (Basel). 2022 May 31;11(11):1488. doi: 10.3390/plants11111488 (PMC9183009; doi:10.3390/plants11111488)
Supplement: Supplementary file 1 [file plants-11-01488-s001.zip › plants-1713607-supplementary/plants-1713607-supplementary/Supplementary Files/supplementary figures1-2.pdf]

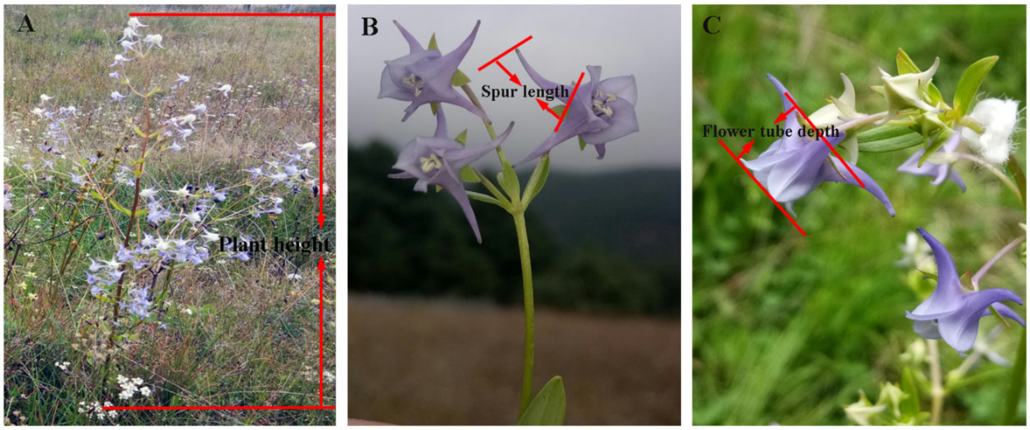

Figure S1. The morphological characteristics for *Halenia elliptica*. A) Plant height, B) spur length, C) flower tube depth.

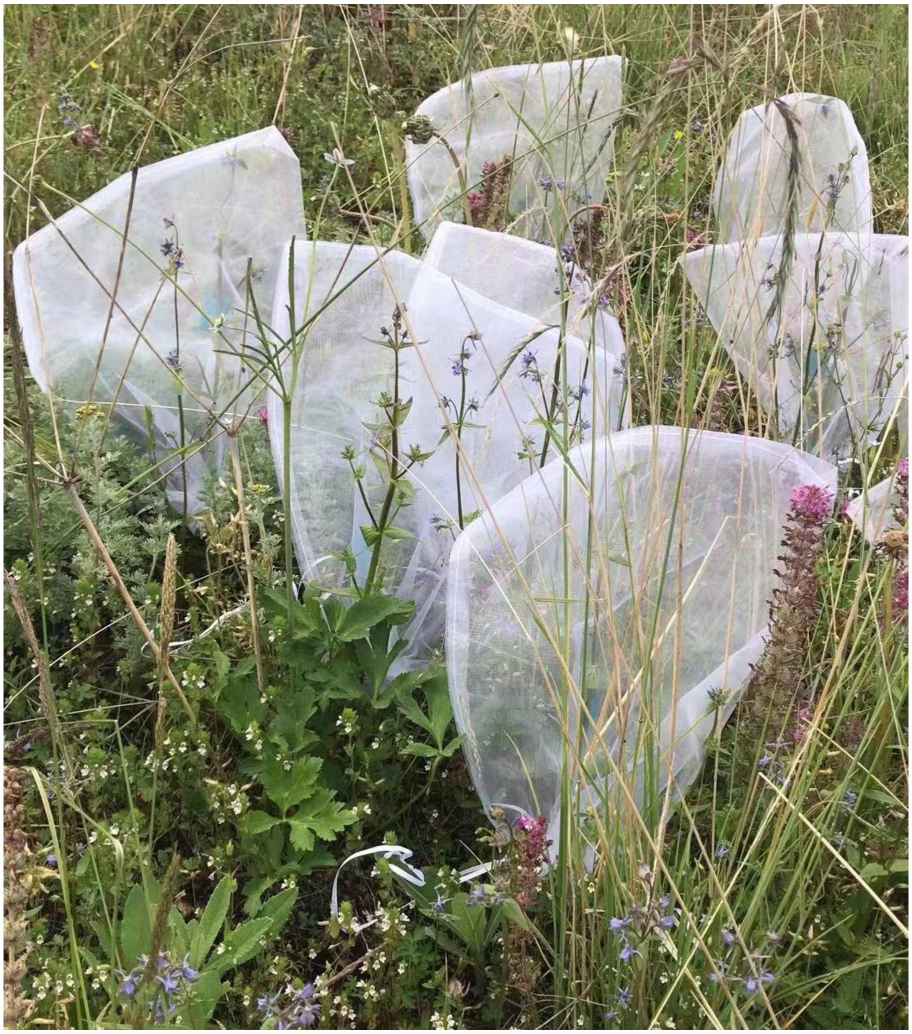

Figure S2. Wrapping the whole plant at the bud stage with mosquito net bags.
